# Risk of Thromboembolism With Eltrombopag: A Case Report of Deep Vein Thrombosis and Bilateral Pulmonary Embolism

**DOI:** 10.7759/cureus.33615

**Published:** 2023-01-10

**Authors:** Ghulam Mujtaba Ghumman, Huda Fatima, Gunbir Singh, Taha Khalid, Mohamed Ayoubi

**Affiliations:** 1 Internal Medicine, St. Vincent Mercy Medical Center, Toledo, USA; 2 Internal Medicine, MedStar Union Memorial Hospital, Baltimore, USA; 3 Hematology and Oncology, St. Vincent Mercy Medical Center, Toledo, USA

**Keywords:** chronic itp, thrombosis, platelets, eltrombopag, thrombocytopenia

## Abstract

Eltrombopag is a second-line therapy for refractory thrombocytopenia from immune thrombocytopenic purpura (ITP). The medication is generally well tolerated but can lead to adverse thromboembolic complications in rare instances. We present a case of lower extremity deep vein thrombosis with bilateral pulmonary embolism in an ITP patient receiving eltrombopag. The patient underwent catheter-directed thrombolysis for pulmonary embolism. Eltrombopag was stopped on discharge, considering the potential cause of venous thrombosis.

## Introduction

Immune thrombocytopenic purpura (ITP) is an autoimmune disease characterized by the rapid peripheral destruction of platelets as well as impaired production of platelets from megakaryocytes within the bone marrow [1]. The initial treatment of ITP includes glucocorticoids and intravenous immunoglobulins (IVIG) to improve platelet count and prevent bleeding [2]. Eltrombopag, a thrombopoietin receptor agonist, is a second-line treatment choice for refractory thrombocytopenia in chronic ITP patients [3]. Eltrombopag is generally well tolerated, but a rare side effect of thromboembolism events has been reported with the medication [4]. We present a case of deep vein thrombosis with bilateral pulmonary embolism related to eltrombopag and discuss the importance of maintaining the desirable range of platelet counts in these patients.

## Case presentation

**Figure 1:**
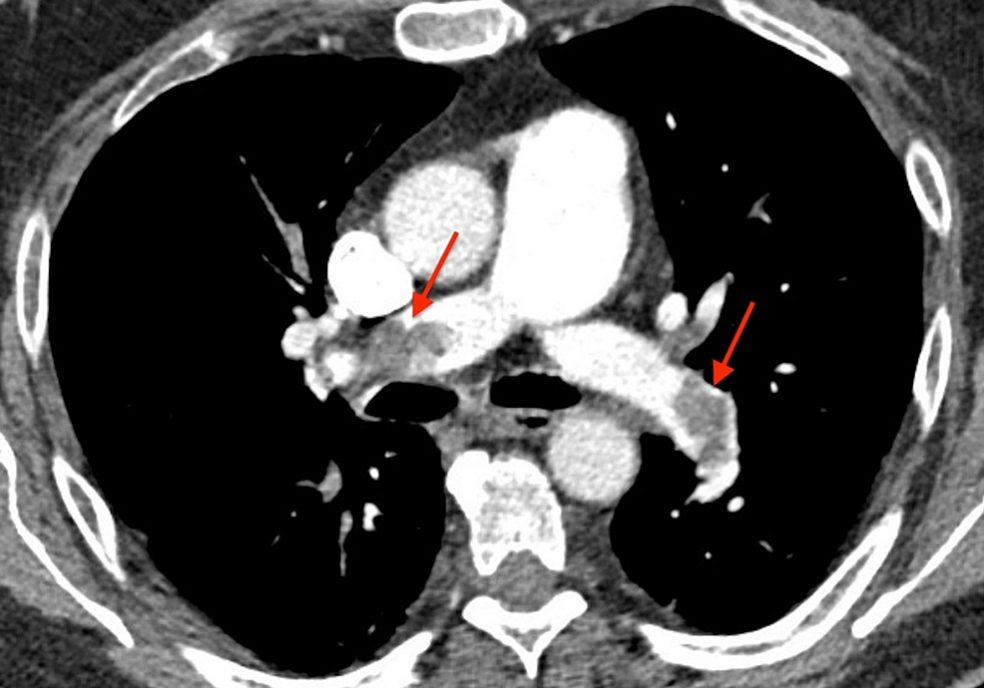
Computerized tomography pulmonary angiogram showing bilateral pulmonary embolism (red arrows)

A 75-year-old male with a past medical history of immune thrombocytopenic purpura (ITP) presented to the emergency department (ED) with chief complaints of shortness of breath and palpitations for five days. He reported that the shortness of breath was mainly exertional and started almost a week ago but worsened over the last two days. He denied cough, orthopnea, chest pain, or fever. The patient also denied taking recent long flights, being bedbound, or having any history of cancer. Physical examination showed a non-toxic appearing man with vital signs: blood pressure of 168/80 mmHg, heart rate of 105 beats/min, respiratory rate of 23 breaths/min, and pulse oximetry oxygen saturation of 96% on 2L nasal cannula oxygen. CT pulmonary angiogram showed a bilateral pulmonary embolism (Figure 1).

Lower extremity venous duplex ultrasound showed acute deep vein thrombosis (DVT) of the right femoral, popliteal, and tibioperoneal veins. The echocardiogram showed reduced right ventricular systolic function with TAPSE (tricuspid annular plane systolic excursion) of 0.8cm and RVSP (right ventricular systolic pressure) of 55 mmHg, while the left ventricular ejection fraction was 51%. The patient had a history of ITP and was initially treated with glucocorticoids and IVIG (intravenous immunoglobulins) with poor response. He subsequently underwent a splenectomy almost three months ago, but he continued to have persistent thrombocytopenia. He was subsequently started (almost three weeks ago) on eltrombopag due to persistent thrombocytopenia even after the splenectomy. A review of previous records showed that his platelet count was 20-30 k/uL before starting eltrombopag. His platelet count on presentation was 173 k/uL and subsequently was noted to be as high as 940 k/uL within a week of hospitalization. The eltrombopag was not resumed. The patient was started on intravenous heparin and underwent successful catheter-directed thrombolysis (CDT) for pulmonary embolism. He was transitioned to warfarin with heparin bridge to target INR 2-3. Eltrombopag was stopped on discharge, and he was advised to follow up with his primary hematologist for close monitoring of platelets and to explore further options for long-term management of ITP.

## Discussion

Eltrombopag is an oral thrombopoietin receptor agonist (TPO-RA) used in treating ITP, which is refractory to steroids, immunosuppressants, and splenectomy if they have an increased risk of bleeding [5]. However, various studies have reported an increased risk of arterial and venous thrombosis associated with the use of eltrombopag [6]. Studies have documented deep vein thrombosis, pulmonary embolism, portal vein thrombosis (PVT), extensive cerebral venous sinus thrombosis, ischemic strokes, and myocardial infarctions as part of the prothrombotic effect caused by the use of TPO-RAs [7-9]. In one study, the thromboembolic events recorded during the three-year follow-up exceeded those experienced by the general population, affecting 5% of patients [7]. In one disproportionality analysis, the median time from the first TPO-RA exposure to thrombosis was recorded to be 76.6 days [10].

Although chronic ITP can be a risk factor for thrombosis, it has been seen that the use of TPO-RA in these patients can lead to adverse effects of thromboembolism, and monitoring for these complications is essential. This has been suggested by some studies where TPO-RAs were used instead of immunosuppressants in chronic ITP patients [11]. A pooled analysis of the four trials to assess the risk of thrombosis in ITP patients showed only a slightly (statistically significant) increased risk of venous thromboembolism in chronic ITP patients (not exposed to TPO-RAs). Some of those trials also showed an increased risk for arterial thrombosis, but statistical significance was not reached (in three out of four trials) [12]. A multicenter cohort study by Ruggeri et al. reported that the incidence of thrombosis in ITP patients was not higher than the acceptable threshold. They concluded that venous and arterial thrombosis is not a frequent complication of ITP, and these patients wouldn't need to be monitored for thrombosis except in elderly patients and those who have undergone splenectomy [13].

The introduction of TPO-RAs for the treatment of refractory ITP raised the issue of thrombosis. An interim analysis on the safety and efficacy of eltrombopag in 299 patients by Saleh et al. reported that 5% of the patients developed thromboembolic events [7]. Rodeghiero et al.'s pooled analysis of 13 clinical trials, including 653 patients who received romiplostim, showed a 6% risk of thrombosis, with 75% of those thromboembolic events occurring during the first year of treatment [14]. These studies on eltrombopag and romiplostim suggested that thrombotic events can occur with low, normal, or high platelet levels. These studies also showed that the annualized risk of venous thromboembolism was four to five times higher and that of arterial thrombosis two times higher with TPO-RA treatment than in an ITP population not exposed to TPO-RAs.

The exact pathogenesis of increased thrombosis with TPO-RA has yet to be well established. Although there has been reported an increase in many coagulation activation markers, for example, D-dimer, thrombin generation, prothrombin fragment, and plasminogen activator inhibitor-1 (PAI-1) in ITP patients, no further activation of coagulation markers has been observed after initiation of TPO-RAs [15]. However, a study published in 2019 reported increased PAI-1 levels in patients treated with TPO-RAs that could lead to the formation of clots resistant to fibrinolysis. It also showed an increase in the microparticle-associated phosphatidylserine procoagulant activity, along with the levels of soluble P-selectin and basal exposure of P-selectin in quiescent platelets in TPO-RAs treated patients, putting them at risk of thrombosis [16].

The target platelet count for patients on eltrombopag should be 30-50 k/μL, which is an optimal balance between bleeding versus thrombotic risk [17]. Achieving a platelet count of 50 k/μL at any point during the treatment is considered a good response to eltrombopag, which as per studies, can mostly be achieved in two weeks [18]. For outpatient clinical practice, the primary goal is to monitor the platelet count until stable counts are achieved to prevent the generation of a thrombus [17]. For eltrombopag, it is recommended to target a platelet count of 50 k/μL, decrease the dosage when platelet counts are above 150 k/μL, and cease treatment if platelet counts are greater than 250 k/μL [17].

## Conclusions

Our case adds to the existing literature of recorded thromboembolic events following the use of eltrombopag; it emphasizes the current underreporting of adverse events associated with TPO-RA use. We also wish to draw attention to the importance of close outpatient follow-up with regular monitoring of platelet counts and keeping a close eye on other thromboembolic risk factors on an individual case basis when prescribing these medications. The subsequent management of ITP in these patients who have had a thromboembolic episode while on TPO-RAs can be challenging.
